# Supporting Breast Milk Expression During Mother–Infant Separation: A Scoping Review of Interventions, Technologies, and Service Delivery Models

**DOI:** 10.3390/healthcare14193297

**Published:** 2026-10-03

**Authors:** Ana Catarina Silva, Sara Palma, Mónica Antunes

**Affiliations:** 1 Escola Superior de Enfermagem São João de Deus, Universidade de Évora, 7000-811 Évora, Portugal; ana.carita.silva@ulsaale.min-saude.pt; 2 Unidade Local de Saúde do Alentejo Central, 7000-811 Évora, Portugal; 3 Centro de Investigação, Inovação e Desenvolvimento em Enfermagem de Lisboa (CIDNUR), Escola Superior de Enfermagem da Universidade de Lisboa, 1600-190 Lisbon, Portugal; sasapalma@enfermagem.ulisboa.pt; 4 CrossI&D Lisbon Research Center, Escola Superior de Saúde da Cruz Vermelha Portuguesa–Lisboa, 1300-125 Lisbon, Portugal; 5 Centre for Interdisciplinary Research in Health (CIIS), Faculty of Health Sciences and Nursing, Universidade Católica Portuguesa, 1649-023 Lisbon, Portugal

**Keywords:** breast milk expression, lactation, mother–infant separation, neonatal intensive care, health services, nursing quality, midwifery, scoping review

## Abstract

Background/Objectives: Mother–infant separation can interrupt direct breastfeeding while lactation is being established. We mapped recent evaluations of strategies supporting milk expression during actual or anticipated separation. Methods: Following JBI guidance and PRISMA-ScR, we searched four databases for English-, Portuguese- or Spanish-language reports published in 2021–2026. Eligible sources evaluated a deliberately implemented technique, technology, equipment-access strategy, professional-education initiative or service change. One reviewer screened and charted the studies; a second reviewer verified decisions and charting, with disagreements resolved by consensus. Results: Twenty-nine reports representing 27 operational study families were included: 12 randomised, three non-randomised controlled and 12 uncontrolled, historical-comparison or descriptive studies. Digital and sensory approaches often improved anxiety, connection or acceptability, whereas effects on milk volume varied. Technique and equipment comparisons were inconsistent. Service changes were repeatedly associated with improved process outcomes, but all five studies in this domain used uncontrolled designs. Conclusions: No expression method was consistently superior. Early practical assistance, appropriate equipment access, individualised support and continuity across maternity and neonatal services appear important, but heterogeneous designs, short-term outcomes and uncontrolled evaluations limit conclusions about effectiveness.

## 1. Introduction

Separation after birth can make direct breastfeeding impossible or difficult at the point when lactation is being established. This is common after preterm birth, neonatal illness, admission to neonatal intensive care and maternal illness. When direct feeding cannot take place, expression is often the route by which lactation is initiated and the infant receives the mother’s own milk. Guidance for small, sick and preterm newborns therefore includes support for milk expression and safe handling of expressed breast milk [1]. In this review, mother’s own milk identifies milk produced by the infant’s mother, and expressed breast milk identifies milk removed by hand or pump. Human milk is retained where a reported feeding outcome includes both mother’s own milk and donor milk. Outcome terminology follows the source report so that these distinct measures are not treated as interchangeable.

The evidence on expression during separation is diverse. Some studies compare hand expression and pumps, while others test relaxation, sensory contact, digital tools, access to equipment, staff education or changes in the organisation of care. These approaches do not act on the same problem, and they do not measure the same outcomes. A woman may find an intervention reassuring without producing more milk, while a method that increases short-term volume may not improve breastfeeding after discharge. Treating these outcomes as interchangeable can obscure what the evidence actually shows.

Breast milk expression during separation is a health-service and nursing quality issue because support starts in maternity care and continues while responsibility shifts to neonatal services. Timely, understandable and actionable information, staff competence, equipment access and continuity determine whether women can implement recommended practices under stressful conditions. The International Confederation of Midwives includes breastfeeding support within the essential competencies for midwifery practice [2]. However, an intervention can be relevant to nursing or midwifery without having been delivered by those professionals. This review therefore distinguishes direct professional evidence from evidence generated in lactation specialist, multidisciplinary or technology-based settings.

Earlier systematic reviews examined methods of milk expression [3] and interventions to promote breastfeeding for infants admitted to neonatal units, including professional support and organisation of care [4]. These syntheses remain relevant to established techniques. The present review provides a time-bounded map of evaluations published in 2021–2026 across expression techniques, equipment, digital and sensory support, professional education and service delivery. Its contribution is to identify what has been evaluated in the recent literature, how support has been delivered, and which outcomes have been examined across these domains. It does not replace earlier reviews or attempt to rank all available expression methods. The review aimed to map deliberately implemented and evaluated support during mother–infant separation within this publication window, and to examine the implications for health-service delivery, nursing quality and midwifery care.

## 2. Materials and Methods

### 2.1. Design and Protocol

This scoping review followed Joanna Briggs Institute (JBI) methodological guidance and the Preferred Reporting Items for Systematic Reviews and Meta-Analyses extension for Scoping Reviews (PRISMA-ScR) [5,6]. The protocol was registered on the Open Science Framework (https://osf.io/ehju5/overview) on 3 July 2026. The database searches were initially run on 3 May 2026, before registration, and a final search update was completed on 9 August 2026. During full-text assessment, it became apparent that the original Concept criterion was too broad for the review question because it admitted observational studies of expression trajectories and descriptive studies of experience in which no support intervention had been evaluated. A dated amendment was registered on 10 August 2026, after full-text assessment of the 69 reports retrieved at that stage and before initial data charting and synthesis. The amendment narrowed the Concept to deliberately implemented and evaluated interventions, technologies, access strategies, educational programmes or service changes. It was applied retrospectively and uniformly to all reports that had met the original eligibility criteria, leading to 34 additional exclusions at that stage. Additional retrieval and record-verification efforts in September 2026 yielded five previously unavailable reports and two further reports outside the preserved database result set. The final Concept criterion and the same reviewer-verification procedures were applied to all seven reports; four were included and three were excluded. Because the amendment was made after the initially retrieved full texts had been read, it was not blind to their content. However, the criterion concerns the presence of an implemented and evaluated support intervention, not the direction, magnitude or statistical significance of the findings.

The review question, addressed within the 2021–2026 publication window, was as follows: What interventions, technologies and service delivery models have been evaluated to support breast milk expression during actual or anticipated separation of mother and infant, what outcomes have been reported, who delivers them and what are the implications for health-service delivery, nursing quality and midwifery care?

### 2.2. Eligibility Criteria

Eligible studies involved mothers expressing milk for preterm, low birth weight, sick or hospitalised newborns, or other situations in which direct feeding at the breast was prevented or materially limited by clinical separation. Anticipatory support was eligible when separation or delayed direct breastfeeding was expected. The final Concept required an identifiable intervention, programme, technology, equipment or access strategy, professional education initiative or service change intended to initiate, facilitate, maintain or improve milk expression, with an expression-related process, outcome or experience that was central or separately analysable.

Primary empirical studies and evaluated quality-improvement or audit reports in English, Portuguese or Spanish were eligible. The publication window was 2021–2026, with the final database search on 9 August 2026. The 2021 lower limit was a pragmatic scope decision intended to provide a bounded map of contemporary evaluations within the clinical-policy context established by the 2020 WHO/UNICEF guidance for small, sick and preterm newborns [1] and to complement, rather than duplicate, earlier syntheses. The Cochrane review of expression methods searched to 21 March 2016 [3], the neonatal breastfeeding-support review searched to February 2008 [4], and the neonatal quality-improvement review searched to 15 January 2021 [7]. These sources informed the historical and clinical context but did not establish the date restriction; nor was it assumed that interventions published after 2020 had been developed in response to the WHO/UNICEF guidance. The boundary is not a clinical threshold, and the review should be read as a map of recent evaluations rather than a comprehensive history or a formal update of an earlier review. Relevant earlier primary studies, particularly on techniques and equipment, may therefore be outside the review window; this limitation is addressed in Section 4.4.

Observational studies describing natural variation in timing, pumping frequency, milk volume, determinants or predictors were excluded when no support intervention was evaluated. Qualitative or descriptive studies were excluded when they reported experiences, barriers or facilitators without evaluating a specific implemented strategy. Reviews, protocols, registries, commentaries, conference-only reports and unevaluated practice descriptions were also excluded. Eligibility was not restricted by professional group because relevant interventions could be delivered by midwives, nurses, lactation specialists, multidisciplinary teams or through self-directed technologies.

### 2.3. Search and Study Selection

PubMed/MEDLINE, CINAHL Complete, Web of Science Core Collection and the Cochrane Library were searched. Search strategies combined controlled vocabulary with free-text terms for breast milk expression, lactation and clinical situations associated with separation, including neonatal intensive care, prematurity and low birth weight. Professional role terms were not required because the review was not limited to interventions named midwife- or nurse-delivered. Intervention design terms were also not mandatory because intervention eligibility was determined during screening. Searches were first run on 3 May 2026, and a final database search update was completed on 9 August 2026. The strategies and yields recorded in the review log at the time of searching are reported in Supplementary File S1.

**Figure 1:**
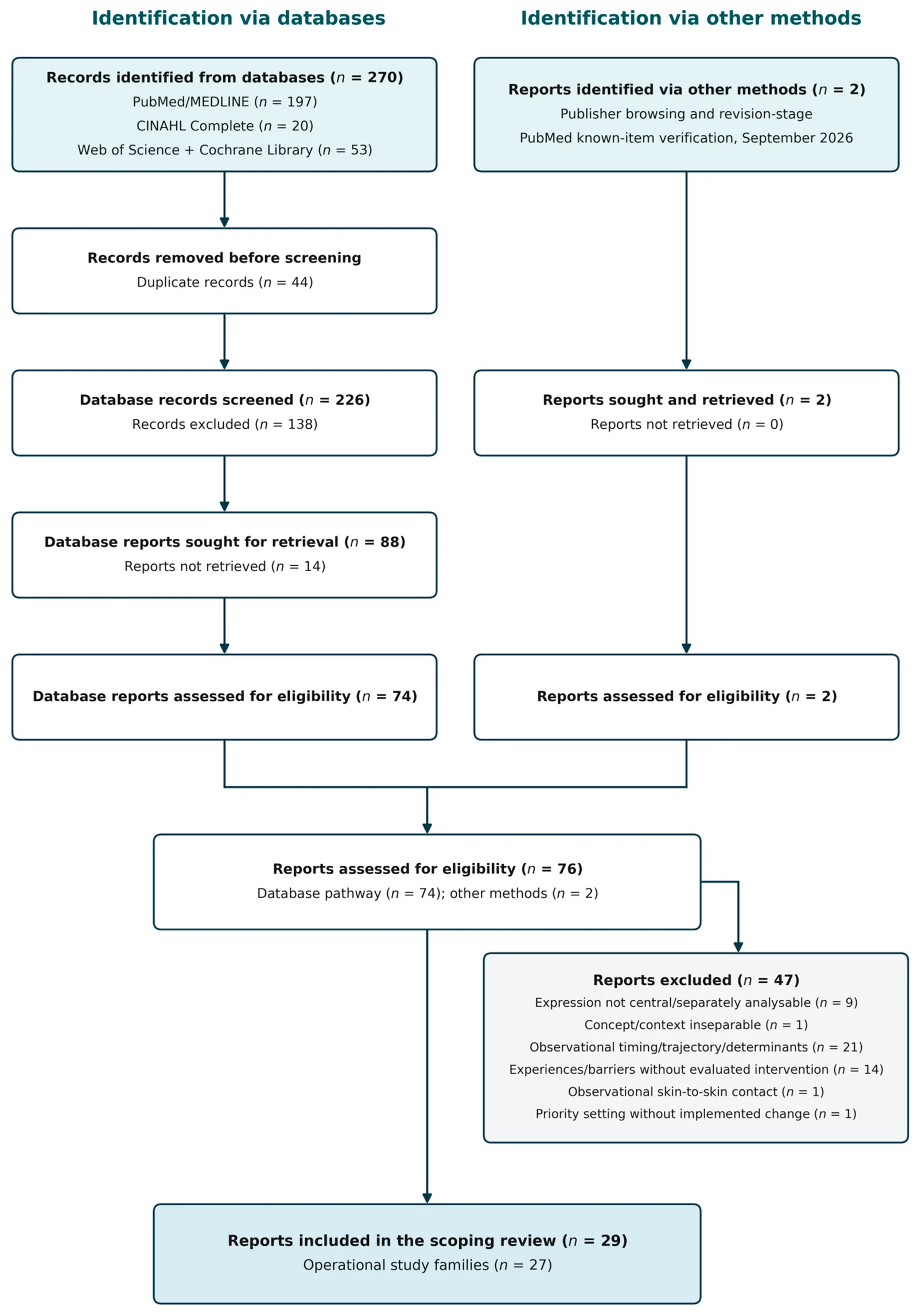
PRISMA-ScR flow diagram showing identification via databases and other methods, screening, full-text assessment and inclusion of 29 reports representing 27 operational study families.

The contemporaneous search records documented 270 database records: 197 from PubMed/MEDLINE, 20 from CINAHL Complete and 53 from Web of Science and the Cochrane Library combined. After removal of 44 duplicates, 226 database records were screened by title and abstract. At the original selection stage, 134 records were excluded, 92 reports were sought, 23 reports were unavailable and 69 underwent full-text assessment. Additional retrieval efforts in September 2026 obtained five of the previously unavailable reports through authenticated B-on access: two met the final eligibility criteria and three were excluded. The indexed abstracts of the 18 still-unresolved reports were then rechecked against the final Concept criterion. Four clearly described non-interventional observational predictor or trajectory studies, or a descriptive service survey, and were reclassified as title/abstract exclusions. The reconciled database pathway therefore comprises 138 records excluded after title/abstract screening, 88 reports sought, 14 reports not retrieved and 74 reports assessed in full text. Two further eligible reports were identified outside the preserved database result set during additional retrieval and record-verification efforts in September 2026 and are reported separately under identification via other methods. In total, 76 full texts were assessed (74 from the database pathway and two identified via other methods), 47 were excluded and 29 reports were included. Supplementary File S4 preserves the retrieval set and documents the five recovered reports, four abstract-stage exclusions, 14 reports that remain unavailable and two additionally identified reports. Figure 1 presents the two identification pathways separately.

A.C.S. performed the initial title and abstract screening and full-text eligibility assessment. All provisional eligibility classifications were then jointly reviewed and confirmed by A.C.S. and M.A. Uncertain or discordant decisions were resolved by discussion and, where needed, re-examination of the available report or indexed abstract until consensus was reached. The same procedure was applied to the reports obtained or identified through additional retrieval efforts in September 2026. During revision-stage reconciliation, the indexed abstracts of the 18 reports that remained unavailable were rechecked against the final Concept criterion; four records were sufficiently clear to exclude at the title/abstract stage and 14 remained potentially eligible without a full-text decision. The final included, excluded and non-retrieved lists were confirmed jointly. This was a consensus-validation process rather than independent blinded duplicate screening.

### 2.4. Data Charting and Study Linkage

A structured charting form captured the study label and citation, publication year, intervention, design and sample, context and population, intervention dose and timing, comparator, provider or mode of delivery, expression-related outcomes, other outcomes, key findings, effect pattern, implementation or acceptability, main limitations, implications for nursing and midwifery, and source identifier. These variables correspond to the column headings in Supplementary File S2. A.C.S. charted the data from the included reports and M.A. subsequently verified the completed charting form, including reports obtained through the additional September 2026 retrieval efforts. Discrepancies were resolved by discussion and re-examination of the source report. Charting was therefore verified by a second reviewer but was not undertaken independently in duplicate.

Two linked investigations each generated two reports. Hoyt-Austin et al. [8] and Hoyt-Austin et al. [9] report complementary quantitative and qualitative findings from the FamilyLink videoconferencing trial. Tanaka and Horiuchi [10,11] report the same education programme involving 36 nurse-midwives. Parker et al. [12] and Magalhães et al. [13] belong to the same biomarker-informed messaging research programme. They are presented as separate report groups because the available descriptions did not allow participant overlap to be determined. Accordingly, the 29 included reports are represented as 27 operational study families rather than as 27 confirmed independent participant samples.

### 2.5. Synthesis

Because the studies varied substantially in design, intervention, comparator and outcome definition, findings were synthesised descriptively rather than pooled. Interventions were grouped into three domains: digital, sensory and behavioural support; expression technique, equipment and access; and professional, organisational and quality improvement support. Milk volume, process outcomes, maternal experience and breastfeeding continuation were interpreted separately. Findings from randomised and non-randomised controlled studies were distinguished from those of uncontrolled designs, including single-group, before-and-after, audit, service evaluation, quality improvement and usability studies, so that favourable results from uncontrolled designs were not presented as evidence of effectiveness. No formal risk of bias appraisal was undertaken because the purpose of the scoping review was to map the range and characteristics of evaluated interventions rather than estimate a pooled effect.

## 3. Results

### 3.1. Study Selection and Characteristics

Of the 76 reports assessed in full text, 47 were excluded. Ten did not meet the original eligibility criteria: nine did not examine milk expression as a central or separately analysable component and one combined concept and context in a way that could not be separated. Under the final Concept criterion, 37 reports were excluded because they did not evaluate an implemented intervention or service strategy. These comprised 21 observational studies of timing, trajectory or determinants, 14 studies of experiences or barriers without an evaluated intervention, one observational study of skin-to-skin contact and one priority-setting study without an implemented change. Twenty-nine reports were included, representing 27 operational study families.

The included studies ranged from randomised trials and quasi-experimental studies to usability studies, service evaluations, audits and quality improvement projects. Twelve study families used randomised designs, three used non-randomised controlled designs, and twelve used uncontrolled, historical-comparison or descriptive designs. All five study families in the professional, organisational and quality improvement domain used uncontrolled designs. Ten study families evaluated digital, sensory or behavioural support; twelve evaluated expression technique, equipment or access; and five evaluated professional, organisational or quality improvement support (Table 1).

### 3.2. Digital, Sensory and Behavioural Support

Ten study families evaluated digital, sensory or behavioural approaches. The FamilyLink crossover trial found that videoconferencing with the hospitalised premature infant improved the mother’s experience of expressing milk, but did not increase milk volume or pumping efficiency [8,9]. The multicentre EXPRESS trial also found no significant increase in expressed milk from a 12 min audio relaxation intervention, although most users who evaluated the recording described it as relaxing [18]. These studies show that an intervention can improve the experience of separation without producing a measurable change in milk volume.

Other sensory approaches produced short-term physiological effects. Shafaati Laleh et al. [14] reported greater milk volume on several study days when mothers watched a video of their infant and smelled an infant-scented hat, together with lower anxiety and stress. Eker et al. [17] also reported lower anxiety and higher milk volume after brief virtual reality sessions. The multicomponent VRBaby-Pump application improved the breast milk expression experience and produced higher recorded milk volumes than training and reminders alone during the first 72 h [15]. The single-centre follow-up was short, and the virtual environment, wearable pump-toy, chatbot and additional intervention contact prevent attribution to any one component. These findings are promising but require replication.

Digital tools were also used for monitoring and continued support. The Neo-MILK application had good usability and was frequently used for milk volume recording, but the study was not designed to test effectiveness on milk production [16]. Two pilot studies of biomarker-informed mobile messaging suggested possible benefits for pumping behaviour or lactation duration, although effects on milk volume were limited or imprecise [12,13]. A WeChat programme was associated with earlier coming to volume and higher milk volume during a later pandemic period, but other service changes occurred at the same time [19]. Early education combined with proactive telephone support was associated with higher expressed milk volume in the first week and longer exclusive breastfeeding duration in a quasi-experimental study [20].

### 3.3. Expression Technique, Equipment and Access

The studies of expression technique and equipment did not identify one method that was consistently superior. A randomised trial comparing Marmet hand expression with a manual pump found no significant difference in daily or cumulative milk volume or exclusive breast milk feeding [26]. In contrast, a smaller quasi-experimental study favoured the Marmet technique [27], while two randomised trials reported higher milk volume with an electric pump than with hand expression [28,29]. In Reddy and Bellad [29], seven-day cumulative volume was higher with electric pumping in both intention-to-treat (667.2 versus 507.3 mL; *p* < 0.001) and per-protocol analyses (747 versus 715 mL; *p* = 0.01); direct breastfeeding at discharge was also reported more often (14 versus 8; *p* = 0.02), although the absolute numbers were small and the one-page report provided limited methodological detail. Taken together, these findings are conflicting rather than evidence of a universal hierarchy of methods.

Some adjunctive techniques increased immediate or session-specific milk yield. Power pumping increased milk expressed during the intervention session but did not improve exclusive breastfeeding at discharge [23]. Back massage before expression increased immediate milk volume in a small controlled study [30]. In a three-arm randomised trial of 36 mothers, back or breast massage produced no between-group difference during the first two days; breast massage exceeded control on day 3 and both massage groups had higher reported volumes on days 4–7 [24]. By day 7, however, only 3, 5 and 5 participants remained under observation in the back-massage, breast-massage and control groups, respectively, which limits interpretation. Anderson et al. [25] tested hand expression with breast massage within the first hour after preterm birth but did not demonstrate clinical superiority over expression within six hours. The pilot also identified practical barriers to very early implementation in the birth setting.

Access to equipment and materials appeared important even when causal inference was limited. A low-cost hospital-grade pump hire programme was highly acceptable and mothers reported that it enabled them to provide expressed milk when cost might otherwise have been a barrier [21]. A standardised colostrum kit containing information and collection materials was associated with a sustained reduction in median time to first colostrum, from 35 h before implementation to 8 h in the latest cohort [22]. A prospective evaluation of a compression and vacuum pump showed favourable usability and volume attainment but had no comparator [31]. In a fixed-order feasibility study, 49 of 50 mothers successfully pumped during kangaroo mother care and continued skin-to-skin contact; session milk volume was comparable with routine pumping, while kangaroo-care duration was longer on the intervention day [32]. The absence of randomised order, the single-session assessment and the convenience sample preclude conclusions about effectiveness.

### 3.4. Professional, Organisational and Quality Improvement Support

Five study families evaluated staff education, dedicated lactation expertise or changes in service organisation. All five used uncontrolled designs, and the most consistently reported changes concerned process outcomes. Boone et al. [33] reported lower pumping discontinuation, higher pumping frequency and greater provision of the mother’s own milk at discharge after a multicomponent quality improvement programme. Quan et al. [34] increased full breast milk feeding on day 7 from 10% at baseline to 80% after introducing a pathway that combined education, early expression support, pump selection, transport and storage guidance and kangaroo care.

Two other service initiatives improved the timing of early support. Minj et al. [35] increased receipt of expressed breast milk within 24 h from 20% to more than 80% and reduced the median time to first expressed milk from 32.5 to 17 h. Following the introduction of a dedicated neonatal lactation specialist, Conboy and Chacko [36] reported increases in breast milk feeding at discharge, assistance with first hand expression within six hours and colostrum availability within six hours. These studies used before-and-after or audit designs, so secular changes cannot be excluded; none included a concurrent control group. The consistency of these process improvements therefore indicates a recurring direction of change across settings, not a higher level of evidence for effectiveness.

The education programme reported by Tanaka and Horiuchi [10,11] provides the clearest evidence directly involving midwives. A 60 min programme improved nurse-midwives’ knowledge, attitudes and implementation of several expression care practices. The linked analysis identified gaps in knowledge retention and the need for practical training. The maternal subgroup was very small and did not show measurable changes in initiation, frequency or volume of expression, so improved professional knowledge cannot yet be assumed to improve lactation outcomes.

### 3.5. Patterns Across Outcomes and Relevance to Health-Service Quality

The studies measured different types of outcomes and these did not move together consistently. Milk volume, timing of expression, pumping continuation, maternal anxiety, comfort, feeding at discharge and professional knowledge should therefore be interpreted as separate outcomes. FamilyLink improved the experience of expression without increasing milk volume, while power pumping increased session yield without improving exclusive breastfeeding at discharge. The short-term milk-volume gains reported in the VRBaby-Pump and massage trials [15,24] do not establish sustained lactation or feeding benefits. Pumping during kangaroo mother care was feasible and maintained comparable session volume in one uncontrolled study, but the longer kangaroo-care duration cannot be attributed confidently to the intervention [32]. Service programmes often showed their clearest reported changes on timing, continuity and availability of the mother’s own milk rather than on a single physiological measure.

Most interventions were not delivered exclusively by nurses or midwives. Evidence came from neonatal nursing, lactation specialist, multidisciplinary and technology-based services. The review therefore treats relevance to nursing and midwifery as an interpretation of where the evidence can inform practice, not as evidence that the intervention has already been shown to be effective when delivered by either profession. Table 2 summarises the main pattern across the three domains.

## 4. Discussion

### 4.1. Principal Findings

This review did not identify a single expression method that consistently produced better outcomes [26,27,28,29]. Instead, the evidence points to three different ways of supporting women when direct breastfeeding is interrupted: making the expression experience less distressing, improving the practical means of removing milk, and organising services so that help is available early and continues for as long as it is needed. Study designs and outcomes differed across these areas, and the review did not formally rank the certainty of evidence.

Digital and sensory interventions often improved how women felt during expression, but physiological effects were less consistent [8,9,14,15,17,18]. This is clinically relevant in its own right. A mother who feels calmer or more connected to her infant may value the intervention even if milk volume does not change. At the same time, these findings argue against using anxiety reduction as a proxy for improved lactation. The same caution applies in the opposite direction. An increase in milk obtained during one pumping session or during the first postpartum days does not necessarily translate into sustained breastfeeding or a better maternal experience [15,23,24].

The technique studies were particularly inconsistent. One small quasi-experimental study favoured hand expression [27], two randomised trials favoured electric pumping [28,29], and one larger randomised trial found no difference between hand expression and a manual pump [26]. Small massage studies reported immediate or short-term volume gains [24,30], but intensive researcher delivery, small samples and attrition limit transferability. A feasibility study suggested that electric pumping can be integrated with kangaroo mother care without reducing session milk volume [32], but its fixed-order, single-observation design does not establish effectiveness. This pattern suggests that clinical advice should not be built around a universal hierarchy of methods. The better question may be whether the chosen method and delivery arrangement are effective enough for the infant’s clinical needs, comfortable and sustainable for the woman, and supported by appropriate equipment and follow-up.

A recurring direction of process change was reported after service changes [22,33,34,35,36]. Programmes that combined early information, practical help, equipment, repeated contact, monitoring and clear responsibility for lactation support were repeatedly associated with better process outcomes. These studies were all uncontrolled quality improvement projects, before-and-after evaluations or audits, so the consistency of their findings reflects a recurring direction of process change rather than causal evidence comparable to that of randomised trials. These evaluations identify delays, equipment access and continuity of responsibility as potential targets for service improvement.

### 4.2. Comparison with Earlier Evidence and International Guidance

The findings on expression technique should be considered alongside the Cochrane review of methods of milk expression, which searched the Cochrane Pregnancy and Childbirth Group’s Trials Register on 21 March 2016 [3]. That review compared hand expression and manual, battery and electric pumps, as well as adjunct and protocol strategies. Across heterogeneous outcomes, it found no clear preference for a single pump type and reported that some lower-cost methods or adjuncts were comparable with, or occasionally favourable to, large electric pumps for specific outcomes [3]. The timing of initiation was examined as a separate protocol question rather than as a device comparison. The review concluded that the most suitable method may depend on time since birth, the purpose of expression and individual circumstances [3]. The conflicting technique comparisons and small massage trials identified in the present review [24,26,27,28,29] likewise do not establish a single superior method. Small samples and heterogeneous interventions limit the generalisability of both the earlier and recent evidence.

The service-level findings are broadly consistent with earlier evidence. Renfrew et al., whose searches extended to February 2008, included interventions involving organisation of care, professional education and support, as well as expression methods and mother–infant contact [4]. The review reported potentially beneficial approaches while noting variation in study design and quality. The recent service evaluations mapped here [22,33,34,35,36] add examples of equipment provision, dedicated support and coordinated care, but their uncontrolled designs limit causal interpretation.

The findings can also be read against current international guidance. The WHO/UNICEF guidance for small, sick and preterm newborns includes support for milk expression, safe handling of expressed breast milk and the minimisation of separation between mothers and infants [1]. ABM Clinical Protocol #35 recommends maintaining milk removal at approximately the usual feeding frequency during separation, regards a hospital-grade double electric pump as the preferred means when pumping fully replaces breastfeeding, and presents hand expression as an option when the infant or a pump is unavailable; it also emphasises keeping mother and child together where possible, skilled lactation support, equipment access and safe milk storage [37]. The interventions identified in this review that improved equipment access, early expression support and continuity of care [21,22,33,34,35,36], together with the feasibility of pumping during kangaroo mother care [32], are concordant with these priorities. Because the supporting studies were mostly uncontrolled, they illustrate feasibility and process change rather than confirm effectiveness.

Fang et al. also synthesised neonatal quality improvement interventions, searching to 15 January 2021 [7]. Their 16 studies comprised 12 uncontrolled before-and-after studies, three controlled before-and-after studies and one interrupted time series. They reported favourable feeding outcomes, although heterogeneous intervention bundles and predominantly non-randomised designs constrain attribution. This earlier service literature reinforces the need to distinguish recurring process improvements from evidence that a particular component causes better lactation outcomes.

### 4.3. Implications for Health Services, Nursing and Midwifery Care

One included study family specifically evaluated education for nurse-midwives [10,11]. This differs from professional involvement in delivering clinical support: Anderson et al. examined early expression in a birth-suite midwifery context [25], and Jiang et al. described obstetric and neonatal nursing within a multidisciplinary lactation pathway [19]. These reports do not isolate a profession-specific effect. The broader implications for nursing and midwifery therefore combine reported professional involvement, the outcomes of multidisciplinary and technology-based interventions, and professional competencies [2]. Potential contributions include timely information, practical assistance, equipment access and continuity between maternity and neonatal services [21,22,34,36].

The findings are also compatible with a less prescriptive approach. Women who are separated from their infants may be recovering from a difficult birth, coping with uncertainty and expressing milk repeatedly while receiving limited feedback from the infant. Care should therefore address comfort, privacy, fatigue, emotional strain and the woman’s own priorities as well as milk volume. Interventions that help connection or reduce distress may be worthwhile even when they do not increase volume [8,9,18], while methods that increase output may not be acceptable or sustainable for every woman [23,25].

Evidence identifying the specific contribution of nurses or midwives remains limited. The Tanaka programme [10,11] reported changes in professional knowledge and practice after education, but its uncontrolled design and outcomes do not establish improved maternal lactation. Future evaluations should report who delivered each intervention component, the amount of support provided, and how maternity and neonatal teams shared responsibility.

### 4.4. Strengths and Limitations

The main strength of this review is the explicit distinction between studies that describe milk expression and studies that evaluate something intended to support it. The protocol amendment is reported transparently, the broader audit trail was retained and linked reports from the same investigation were counted as one study family. Study selection and data charting were subjected to two-reviewer consensus validation, although they were not undertaken as independent blinded duplicate processes. The synthesis also separates physiological, process, experiential and feeding outcomes rather than treating any statistically significant result as evidence of overall breastfeeding effectiveness.

Several limitations remain. Four of the 18 reports that remained unresolved after retrieval were excluded during a revision-stage audit because their indexed abstracts clearly described non-interventional observational predictor or trajectory studies, or a descriptive service survey. Fourteen database-pathway reports were not retrieved, representing 15.9% of the 88 reports sought through database searching; both reports identified via other methods were retrieved. All 14 appear potentially eligible from title and abstract but could not receive a full-text decision. Their absence may therefore have altered the distribution of interventions, designs and outcomes and introduces a risk of availability bias. Supplementary File S4 provides the record-level retrieval inventory and preserves the four abstract-stage decisions separately. ResearchGate requests were submitted for a subset of restricted records, but no response had been received by 25 September 2026. The two eligible reports identified outside the preserved database result set indicate imperfect search sensitivity. For Reddy and Bellad [29], the retained workbooks do not establish whether the record was absent from the original export or excluded during title and abstract screening; it is therefore reported transparently under other methods rather than assigned retrospectively to the database pathway. Potential participant overlap between the two biomarker-informed messaging reports [12,13] limits interpretation of study-family counts. The evidence is heterogeneous, with small trials, quasi-experimental studies, audits and multicomponent quality-improvement programmes. Many studies were conducted in a single centre, and several measured only short-term milk volume. Service bundles and uncontrolled designs prevent reliable attribution of changes to individual components. Professional roles were incompletely reported, limiting profession-specific conclusions.

Search yields for Web of Science and the Cochrane Library were retained only as a combined total, limiting source-specific reproducibility. The focus on recent publications also limits historical completeness. More specifically, the Cochrane expression-methods review searched to 21 March 2016 [3], whereas the present review begins with 2021 publications; primary studies published from 22 March 2016 to 31 December 2020 were therefore not systematically covered within this review framework. Absence from this recent evidence map should not be interpreted as absence from the wider literature. The searches were run before protocol registration, and the Concept criterion was narrowed after the initially retrieved full texts had been read. Although the amendment was documented, applied consistently and concerned intervention presence rather than result direction, its timing provides less protection against review-stage decision bias than fully prospective eligibility criteria. Screening and charting were verified by a second reviewer but were not performed independently in duplicate. These limitations should be considered when interpreting the map.

### 4.5. Priorities for Research

Further research should evaluate clearly described nursing- and midwifery-provided support, including intervention content, timing, frequency and co-interventions. Studies that follow women across maternity and neonatal services are needed because the current evidence suggests that continuity may be as important as the expression method itself. Future trials and implementation studies should measure maternal experience, burden, sleep, recovery and acceptability alongside milk volume and feeding outcomes. Equity and health literacy also require more attention, particularly access to hospital-grade pumps, digital support, language-appropriate information and specialist lactation care. Controlled or stepped implementation designs are needed to test whether the service-level changes that were repeatedly associated with improved process outcomes also improve lactation and feeding outcomes.

## 5. Conclusions

Within the 2021–2026 publication window, 27 study families were mapped and no single expression technique was consistently superior when mothers and infants were separated. Digital and sensory interventions could reduce anxiety or improve the experience of expression; some small short-term trials reported higher milk volume, but effects were not consistent across studies. Technique and device comparisons produced conflicting results, and massage findings came from small trials with important implementation or follow-up limitations. Improvements in process outcomes were most often reported when services provided early practical help, equipment, repeated support and clear continuity of care, although this evidence came from uncontrolled designs and does not yet establish effectiveness. For maternity and neonatal nursing and midwifery services, the main implication is to recognise separation early, provide individualised support and ensure that lactation care continues when responsibility moves between services. More direct evaluation of professionally delivered interventions is needed.

## Figures and Tables

**Table 1:** Characteristics and principal findings of the 27 included study families.

Study	Design, Sample and Population	Intervention	Main Outcomes	Principal Finding
Hoyt-Austin et al. [8]; Hoyt-Austin et al. [9]	Randomised crossover trial; 39 enrolled, with outcome-specific analytic samples (28 for volume/efficiency); linked qualitative report Mothers expressing milk for hospitalised premature infants	Three expression sessions with live videoconferencing and three without.	Milk volume; expression efficiency	Improved expression experience, with no difference in milk volume or pumping efficiency.
Shafaati Laleh et al. [14]	Three-arm randomised controlled trial; *n* = 78 mothers. Mothers of infants hospitalised in neonatal intensive care unit	Infant video with or without an infant scented hat for 5 days.	Daily milk volume	Hat plus video increased milk volume on days 3 to 5 and reduced anxiety and stress. Video alone increased volume on day 5.
Özkaya et al. [15]	Two-arm parallel randomised controlled trial; *n* = 88 (44/group), stratified by parity Mothers within 24 h of preterm birth whose infants were admitted to neonatal intensive care, Türkiye	Breast milk expression training plus the VRBaby-Pump application for 15 min once daily on two days. The application combined a virtual baby room, a wearable pump fitted to a toy baby and a virtual nurse chatbot; both groups received expression reminders.	Breast Milk Expression Experience Scale; milk volume during 0–24, 24–48 and 48–72 h	Expression-experience scores improved (4.31 ± 0.53 vs. 3.28 ± 0.99; *p* < 0.001). Recorded milk volume was higher in the intervention group during 0–24 h (17.50 ± 21.51 vs. 2.98 ± 3.63 mL), 24–48 h (34.68 ± 26.38 vs. 5.23 ± 13.43 mL) and 48–72 h (61.36 ± 63.10 vs. 9.77 ± 24.01 mL); group and time-by-group effects were significant (both *p* < 0.001). Single-centre, 72 h follow-up and the multicomponent intervention limit attribution and generalisability.
Schwab et al. [16]	Cross-sectional usability study; 63 mothers analysed (80 respondents; 341 contacted) Mothers of sick/preterm infants in neonatal intensive care unit, Germany	Web app providing lactation information and milk volume tracking.	Milk volume documentation use; not an effectiveness trial	Good usability and satisfaction. Effectiveness on milk production was not tested.
Eker et al. [17]	Quasi-experimental within-participant study; *n* = 30 Mothers of preterm infants (30 to 37 weeks) admitted to neonatal intensive care unit	Ten-minute virtual reality relaxation before expression for 3 days.	Expressed milk volume	Anxiety decreased and milk volume increased after the intervention in this small nonrandomised study.
Levene et al. [18]	Multicentre randomised controlled trial; *n* = 132 mothers of very preterm infants Very preterm birth (23 + 0 to 31 + 6 weeks), United Kingdom	Twelve-minute audio relaxation intervention.	Highest 24 h expressed milk weight on days 4, 14 or 21	No significant difference in milk volume or anxiety. Most users described the audio as relaxing.
Parker et al. [12]	Randomised controlled pilot; *n* = 51 Pump-dependent mothers of critically ill neonatal intensive care unit infants	Personalised text messages guided by pumping frequency and milk sodium.	Pumping frequency; milk volume; coming to volume; lactation duration	No difference in daily milk volume. Pumping frequency improved on day 5 and lactation duration was nearly 9 days longer.
Magalhães et al. [13]	Randomised pilot trial; *n* = 36 Mothers of critically ill/preterm infants, largely socioeconomically vulnerable	Personalised mobile support using milk biomarker feedback.	Lactation duration; mother’s own milk feeding at 3 months	Pilot estimates favoured ongoing lactation and the mother’s own milk feeding at 3 months, but were imprecise.
Jiang et al. [19]	Comparative period study; 482 mothers/544 preterm infants neonatal intensive care unit during pre-pandemic, early-pandemic and later-pandemic periods, China	WeChat programme with education, recording and access to lactation consultation.	Pumping frequency, maternal milk volume, coming to volume; human milk feeding (mother’s own and donor milk)	The later programme period had higher milk volume from day 4 and earlier coming to volume, although period effects limit causal inference.
Saehoong et al. [20]	Quasi-experimental study; 57 mother–infant dyads (29 intervention, 28 control) Mothers of preterm infants, Thailand	Early breastfeeding education with proactive telephone follow-up.	Total expressed milk volume days 1 to 7; breastfeeding duration	Higher milk volume in the first week and longer exclusive breastfeeding duration. Exclusive breastfeeding at 3 months was also higher.
Sweet et al. [21]	Multi-method service evaluation; 24 mothers/26 infants neonatal intensive care unit mothers experiencing financial hardship; infants <32 weeks and/or <1500 g	Hospital grade pump hire for one dollar per day during admission and home expression.	Ability to provide expressed milk; feeding status at discharge	Highly acceptable. Fourteen of 26 infants were exclusively breast milk fed at discharge, but the study had no control group.
Hellström et al. [22]	Retrospective observational before-and-after cohort; 2814 infants with analysable timing data Infants admitted to neonatal intensive care unit within 1 h of birth	Colostrum kit with hand expression guidance and collection, labelling and transport materials.	Time from birth to first colostrum received	Median time to first colostrum fell from 35 h before implementation to 8 h in the latest cohort.
Kalathingal et al. [23]	Pilot randomised controlled trial; *n* = 78 randomised and *n* = 72 analysed (36/group) Mothers of very-low-birth-weight infants with low milk output on/after postnatal day 14	One daily power pumping session for 7 days in addition to routine support.	Milk volume per pumping session; exclusive breastfeeding at discharge	Greater milk volume in the intervention session on day 7, with no difference in exclusive breastfeeding at discharge.
Kılcı Erciyas and Kavlak [24]	Three-arm single-centre randomised controlled trial; *n* = 36 (12/group) Mothers after preterm birth whose infants received neonatal intensive care, Türkiye	Back massage for 15 min, breast massage for 10 min per breast, or control, initiated within 6 h and repeated at four scheduled times, followed by hand expression.	Daily expressed-milk volume through day 7; state and trait anxiety	No between-group milk-volume difference was reported on days 1–2. Breast massage exceeded control on day 3; both massage groups exceeded control on days 4–7. State anxiety at 6 weeks was lower in both massage groups. Only 3, 5 and 5 participants remained under observation on day 7, and the intensive researcher-delivered protocol limits transferability.
Anderson et al. [25]	Exploratory parallel pilot randomised controlled trial; *n* = 31 Mothers of preterm infants in metropolitan maternity hospital, Australia	Hand expression with breast massage within 1 h compared with standard expression within 6 h.	Milk production	Feasibility targets were not met and no clear clinical superiority was shown. Birth suite implementation barriers were identified.
Dhanawat et al. [26]	Randomised controlled trial; 170 randomised, 137 with 7-day data Mothers of infants born <34 weeks	Marmet hand expression compared with a manual pump.	Daily and cumulative milk volume; exclusive breast milk feeding	No significant differences in milk volume or exclusive breast milk feeding.
Kotsu et al. [27]	Quasi-experimental time-series; *n* = 40 (20 intervention, 20 control) Mothers with infants admitted to neonatal intensive care unit	Marmet technique compared with breast pump expression over 3 days.	Expressed breast milk volume	The Marmet group produced higher short-term milk volume in this small quasi-experimental study.
Aparna et al. [28]	Randomised trial; sample size not stated in the retained charting record Mothers of very-low-birth-weight infants (<1500 g) in neonatal intensive care	Hand expression compared with an electric pump, starting within 24 h.	Daily expressed milk volume	The electric pump group had higher daily milk volume from day 2 to day 10.
Reddy and Bellad [29]	Randomised controlled trial; *n* = 201 (100 electric-pump; 101 manual-expression) Mothers of infants born <37 weeks and weighing <2.5 kg admitted to neonatal intensive care, India	Electric-pump expression versus manual expression, initiated within 1 h after birth; six 10–15 min sessions daily for 7 days.	Seven-day cumulative milk volume; macronutrient composition; feeding type; infant discharge weight	Cumulative milk volume was higher with electric pumping in intention-to-treat (667.2 vs. 507.3 mL; *p* < 0.001) and per-protocol analyses (747 vs. 715 mL; *p* = 0.01). Macronutrient composition was similar; direct breastfeeding at discharge was reported in 14 versus 8 infants (*p* = 0.02).
Aswathi and Soya [30]	Experimental pretest to posttest control-group study; *n* = 60 (30/group) Mothers of neonates admitted to neonatal intensive care unit	Seven to 10 min back massage immediately before milk expression.	Expressed milk volume	The intervention group had a greater immediate increase in milk volume than the control group.
Wight et al. [31]	Prospective descriptive study; 51 enrolled, 36 complete 15-day pumping logs Exclusively pumping mothers of very low birth weight infants in neonatal intensive care unit	Compression and vacuum pump used by mothers expressing exclusively for 15 days.	Pumping frequency; achieved daily milk volume	Most mothers reached prespecified milk volume thresholds and reported favourable usability. There was no comparator.
Parikh et al. [32]	Cross-sectional feasibility study with fixed-order within-participant comparison; *n* = 50 mother–infant dyads Mothers of stable preterm infants older than 7 days, on full gavage feeds of expressed mother’s own milk, India	Day 1: double-sided hospital-grade pumping outside kangaroo mother care. Day 2: pumping during ongoing kangaroo mother care with lactation-nurse assistance.	Feasibility and acceptability; session milk volume; duration of kangaroo mother care	Forty-nine of 50 mothers (98%) expressed successfully and continued kangaroo mother care. Session volume was comparable (40 vs. 50 mL; *p* = 0.544), while mean kangaroo-care duration increased from 2.0 to 2.9 h/session and from 3.9 to 5.7 h/day (both *p* < 0.001). Fixed order and single-session assessment limit causal interpretation.
Boone et al. [33]	Single-centre before-and-after quality-improvement initiative; *n* = 284 mothers (109 baseline; 175 intervention/sustainment), with 320 infants admitted Preterm/very-low-birth-weight infants admitted to neonatal intensive care within 24 h of life	Multicomponent neonatal quality improvement bundle addressing lactation processes and disparities.	Pumping frequency; pumping discontinuation; mother’s own milk at discharge	Pumping discontinuation fell from 49% to 35% and the mother’s own milk at discharge increased from 36% to 58%.
Quan et al. [34]	Longitudinal quality-improvement project; *n* = 180 infants analysed (40 baseline; 140 intervention) Hospitalised late-preterm infants and mothers, China	Integrated education, early expression, pump selection, milk measurement, transport and storage support.	Full breast milk feeding (≥120 mL/kg/day) on day 7; expression processes	Full breast milk feeding on day 7 increased from 10% to 80% and the improvement was sustained.
Tanaka and Horiuchi [10,11]	Single-group before-and-after intervention; *n* = 36 nurse-midwives; linked 2022 secondary analysis Obstetric ward of a Japanese perinatal medical centre; care for mothers of preterm infants	Sixty-minute education programme for nurse-midwives on early expression care.	Care implementation; maternal initiation/frequency/volume explored in very small maternal sample	Knowledge, attitudes and care practices improved. No measurable maternal expression effect was found in the very small maternal subgroup.
Minj et al. [35]	Quality-improvement initiative; *n* = 17 very preterm infants over 3 months Very preterm infants <32 weeks in tertiary neonatal intensive care unit	Counselling, educational materials, frequent visits and telephone reminders across quality improvement cycles.	Receipt of expressed breast milk within 24 h; time to first expressed breast milk	Receipt of expressed milk within 24 h rose from 20% to more than 80% and median time to first milk fell to 17 h.
Conboy and Chacko [36]	Retrospective audit; 156 infants for discharge feeding analysis and 61 for timing sub-audit Infants born <32 weeks in a tertiary neonatal intensive care unit	Dedicated neonatal lactation specialist.	Timing of first assistance with hand expression; colostrum availability; breast milk feeding at discharge	Breast milk feeding at discharge increased from 59% to 85%. Early expression assistance and colostrum availability increased to 92%.

**Table 2:** Synthesis of intervention domains and implications for service quality.

Intervention Domain	Study Families	Pattern of Evidence	Implications for Health Services, Nursing and Midwifery Care
Digital, sensory and behavioural support	10	Experience, anxiety and acceptability often improved, but effects on milk volume varied. Several small sensory and virtual-reality trials reported short-term volume gains, while other randomised studies reported null effects.	These approaches may reduce distress and help women feel connected to their infant. Short-term gains from multicomponent or small trials require replication and should not be presented as reliable sustained effects on milk production.
Technique, equipment and access	12	Comparisons of hand expression and pumps produced conflicting findings. Massage and power pumping sometimes increased immediate or short-term yield, but the massage trials were small. Equipment access and a colostrum kit improved acceptability or timing in uncontrolled evaluations. Pumping during kangaroo mother care was feasible in one fixed-order study.	Method choice should take account of clinical need, comfort, preference and access. Massage may be considered as an adjunct, but the intensive delivery and limited follow-up of the available trials do not establish routine effectiveness.
Professional, organisational and quality improvement support	5	Improvements were repeatedly reported in time to first expression or colostrum, pumping continuation and provision of the mother’s own milk after service redesign or dedicated support. All five studies used uncontrolled designs.	By inference, maternity and neonatal nurses and midwives may contribute by anticipating separation, initiating practical support and ensuring continuity when care moves between services. One study family targeted nurse-midwife education; clinical nursing and midwifery involvement is also reported in other interventions, without isolating a profession-specific effect.

## Data Availability

No new data were created or analyzed in this study. Data sharing is not applicable to this article.

## Supplementary Materials

The following supporting information can be downloaded at: https://www.mdpi.com/article/10.3390/healthcare14193297/s1, Supplementary File S1, database search strategies and contemporaneously recorded yields, with two additionally identified reports reported separately; Supplementary File S2, study-level charting data and included-report bibliography; Supplementary File S3, PRISMA-ScR checklist; Supplementary File S4, final inventory of 14 reports not retrieved, audit of four abstract-stage exclusions, audit of five reports obtained in September 2026, and two reports identified outside the preserved database result set. References [38,39,40,41,42,43,44,45,46,47,48,49,50,51,52,53,54,55,56,57,58] are cited in Supplementary File S4.
