# Supplementary material for: Supporting Breast Milk Expression During Mother–Infant Separation: A Scoping Review of Interventions, Technologies, and Service Delivery Models
Source: Healthcare (Basel). 2026 Oct 3;14(19):3297. doi: 10.3390/healthcare14193297 (PMC13665052; doi:10.3390/healthcare14193297)
Supplement: Supplementary file 1 [file healthcare-14-03297-s001.zip › Supplementary_File_S1_Search_Strategies_FINAL.pdf]

Supplementary File S1

Final database search strategies and verified search yields

Scoping review: Supporting Breast Milk Expression During Mother–Infant Separation: A Scoping Review of Interventions, Technologies, and Service Delivery Models

Search histories and yields were verified in the database platforms on 11 August 2026. Searches were limited to evidence published from 2021 to 2026 in English, Portuguese or Spanish. The 2021 start date was selected to capture contemporary interventions, technologies and service models relevant to current maternity and neonatal practice. Professional role terms and intervention design terms were not mandatory because professional group and intervention eligibility were determined during screening.

Protocol: Open Science Framework, https://osf.io/ehju5/overview (registered 3 July 2026; scope amendment dated 10 August 2026).

1. PubMed/MEDLINE

```
(
  "Breast Milk Expression"[Mesh]
  OR "Breast Feeding"[Mesh]
  OR "Milk, Human"[Mesh]
  OR "Lactation"[Mesh]
  OR "breast milk express*"[tiab]
  OR "human milk express*"[tiab]
  OR "milk express*"[tiab]
  OR "expressed breast milk"[tiab]
  OR "expressed human milk"[tiab]
  OR "expressed milk"[tiab]
  OR "hand express*"[tiab]
  OR "manual express*"[tiab]
  OR "breast pump*"[tiab]
  OR pumping[tiab]
  OR "milk volume"[tiab]
  OR "milk production"[tiab]
  OR "lactation support"[tiab]
)
AND
(
  "Intensive Care Units, Neonatal"[Mesh]
  OR "Infant, Premature"[Mesh]
  OR "Infant, Low Birth Weight"[Mesh]
  OR "Infant, Very Low Birth Weight"[Mesh]
  OR "Infant, Newborn, Diseases"[Mesh]
  OR NICU[tiab]
  OR "neonatal intensive care"[tiab]
  OR "neonatal intensive care unit*"[tiab]
  OR "neonatal unit*"[tiab]
  OR preterm[tiab]
  OR premature[tiab]
  OR VLBW[tiab]
  OR "very low birth weight"[tiab]
  OR "low birth weight"[tiab]
  OR "hospitalized infant*"[tiab]
  OR "hospitalised infant*"[tiab]
  OR "hospitalized newborn*"[tiab]
  OR "hospitalised newborn*"[tiab]
  OR "critically ill infant*"[tiab]
  OR "sick infant*"[tiab]
  OR "mother-infant separation"[tiab]
  OR "mother infant separation"[tiab]
  OR "maternal infant separation"[tiab]
)
AND ("2021/01/01"[Date - Publication] : "2026/12/31"[Date - Publication])
AND (english[Language] OR portuguese[Language] OR spanish[Language])
```

Verified yield: n = 197.

2. CINAHL Complete (EBSCOhost)

```
S1  (MH "Breast Feeding+")
     OR (MH "Lactation+")
```

```
     OR (MH "Human Milk")
     OR TI ("breast milk express*" OR "human milk express*" OR "milk express*" OR
            "expressed breast milk" OR "expressed human milk" OR "expressed milk" OR
            "hand express*" OR "manual express*" OR "breast pump*" OR pumping OR
            "milk volume" OR "milk production" OR "lactation support")
     OR AB ("breast milk express*" OR "human milk express*" OR "milk express*" OR
            "expressed breast milk" OR "expressed human milk" OR "expressed milk" OR
            "hand express*" OR "manual express*" OR "breast pump*" OR pumping OR
            "milk volume" OR "milk production" OR "lactation support")


S2  (MH "Intensive Care Units, Neonatal+")
     OR (MH "Infant, Premature+")
     OR (MH "Infant, Low Birth Weight+")
     OR TI (NICU OR "neonatal intensive care" OR "neonatal unit*" OR preterm OR premature
OR
            VLBW OR "very low birth weight" OR "low birth weight" OR
            "hospitali?ed infant*" OR "hospitali?ed newborn*" OR "critically ill infant*"
OR
            "sick infant*" OR "mother-infant separation" OR "mother infant separation")
     OR AB (NICU OR "neonatal intensive care" OR "neonatal unit*" OR preterm OR premature
OR
            VLBW OR "very low birth weight" OR "low birth weight" OR
            "hospitali?ed infant*" OR "hospitali?ed newborn*" OR "critically ill infant*"
OR
            "sick infant*" OR "mother-infant separation" OR "mother infant separation")


S3  S1 AND S2


Limiters: Published Date 20210101-20261231;
           Language: English OR Portuguese OR Spanish
```

Verified yield: n = 20.

3. Web of Science Core Collection

```
TS=(
  "breast milk express*" OR "human milk express*" OR "milk express*" OR
  "expressed breast milk" OR "expressed human milk" OR "expressed milk" OR
  "hand express*" OR "manual express*" OR "breast pump*" OR pumping OR
  breastfeeding OR "breast feeding" OR lactation OR "human milk" OR
  "milk volume" OR "milk production" OR "lactation support"
)
AND
TS=(
  NICU OR "neonatal intensive care" OR "neonatal intensive care unit*" OR
  "neonatal unit*" OR preterm OR premature OR VLBW OR "very low birth weight" OR
  "low birth weight" OR "hospitalized infant*" OR "hospitalised infant*" OR
  "hospitalized newborn*" OR "hospitalised newborn*" OR "critically ill infant*" OR
  "sick infant*" OR "mother-infant separation" OR "mother infant separation" OR
  "maternal infant separation"
)


Refine: Publication Years = 2021-2026
        Languages = English OR Portuguese OR Spanish
```

Verified yield: n = 53 (Web of Science and Cochrane Library combined; working search log retained the combined total but not the separate count for each source).

4. Cochrane Library Search Manager

```
#1  [mh "Breast Milk Expression"]
#2  [mh "Breast Feeding"]
#3  [mh "Milk, Human"]
#4  [mh Lactation]
#5  ("breast milk express*" OR "human milk express*" OR "milk express*" OR
     "expressed breast milk" OR "expressed human milk" OR "expressed milk" OR
     "hand express*" OR "manual express*" OR "breast pump*" OR pumping OR
     "milk volume" OR "milk production" OR "lactation support"):ti,ab,kw
```

```
#6  #1 OR #2 OR #3 OR #4 OR #5

#7  [mh "Intensive Care Units, Neonatal"]
#8  [mh "Infant, Premature"]
#9  [mh "Infant, Low Birth Weight"]
#10 [mh "Infant, Very Low Birth Weight"]
#11 (NICU OR "neonatal intensive care" OR "neonatal intensive care unit*" OR
     "neonatal unit*" OR preterm OR premature OR VLBW OR "very low birth weight" OR
     "low birth weight" OR "hospitalized infant*" OR "hospitalised infant*" OR
     "hospitalized newborn*" OR "hospitalised newborn*" OR "critically ill infant*" OR
     "sick infant*" OR "mother-infant separation" OR "mother infant separation"):ti,ab,kw
#12 #7 OR #8 OR #9 OR #10 OR #11
#13 #6 AND #12
```

Apply publication-year/date limits corresponding to 2021–2026. Language eligibility (English/Portuguese/Spanish) may be applied during screening if the interface does not provide a reliable language limiter across all Cochrane content types.

Verified yield: n = 53 (Web of Science and Cochrane Library combined; see above).

5. Search and screening summary

PRISMA stage	n
Records identified from databases	270
Duplicate records removed	44
Records screened by title and abstract	226
Records excluded after title and abstract screening	134
Reports sought for retrieval	92
Reports not retrieved	23
Reports assessed in full text	69
Reports excluded after full text assessment	44
Reports included in the intervention-focused synthesis	25
Independent study families represented	23

6. Reporting note

The final database total was 270 records. After deduplication, 226 records entered title and abstract screening. Screening used the review Population, Concept and Context criteria, publication date, language and evidence type. One hundred and thirty-four records were excluded and 92 reports were sought for retrieval. Full-text exclusion reasons are reported in Figure 1 and in the screening audit.
