# Supplementary material for: Supporting Breast Milk Expression During Mother–Infant Separation: A Scoping Review of Interventions, Technologies, and Service Delivery Models
Source: Healthcare (Basel). 2026 Oct 3;14(19):3297. doi: 10.3390/healthcare14193297 (PMC13665052; doi:10.3390/healthcare14193297)
Supplement: Supplementary file 1 [file healthcare-14-03297-s001.zip › Supplementary_File_S3_PRISMA_ScR_Checklist_FINAL.pdf]

PRISMA Extension for Scoping Reviews (PRISMA-ScR) Checklist

Supporting Breast Milk Expression During Mother–Infant Separation: A Scoping Review of Interventions, Technologies, and Service Delivery Models

Reporting guideline: Tricco AC et al. Ann Intern Med. 2018;169:467–473. doi:10.7326/M18-0850

Section/topic	Item	PRISMA-ScR checklist item	Reported	Location in manuscript
TITLE				
Title	1	Identify the report as a scoping review.	Yes	Manuscript p.1: title identifies the report as a scoping review.
ABSTRACT				
Structured summary	2	Provide a structured summary including background, objectives, eligibility criteria, sources, charting methods, results and conclusions, as applicable.	Yes	Manuscript p.1: abstract structured under Problem; Background; Question, hypothesis or aim; Methods; Findings; Discussion; Conclusion.
INTRODUCTION				
Rationale	3	Describe the rationale for the review in the context of what is already known and why a scoping review approach is appropriate.	Yes	Section 1, pp.2–3: heterogeneous interventions and non-equivalent outcomes establish the need to map the evidence.
Objectives	4	State the questions and objectives with reference to key elements such as population, concept and context.	Yes	Section 1, p.3; Section 2.1, p.3.
METHODS				
Protocol and registration	5	State whether a protocol exists, where it can be accessed and any registration information.	Yes	Section 2.1, p.3: OSF registration (https://osf.io/ehju5/overview), date and amendment reported.
Eligibility criteria	6	Specify eligibility characteristics, including years, language and publication status, and provide a rationale.	Yes	Section 2.2, p.4: population, concept, context, evidence types, languages and 2021–2026 window with rationale.
Information sources	7	Describe all information sources and the date of the most recent search.	Yes	Section 2.3, pp.4–5: four databases and verification date (11 August 2026); Supplementary File 1.
Search	8	Present the full electronic search strategy for at least one database, including limits, so it can be repeated.	Yes	Supplementary File 1: full strategies for all four databases and verified yields.
Selection of sources	9	State the process for selecting sources of evidence.	Yes	Section 2.3, pp.4–5: screening, focused refinement and two-reviewer consensus validation reported.
Data charting process	10	Describe charting methods, including calibration or testing, independent or duplicate charting, and confirmation with investigators, as applicable.	Partial	Section 2.4, p.5: structured charting form, fields and linked-report handling reported; independent/duplicate charting and investigator contact are not specified.
Data items	11	List and define all variables for which data were sought and any assumptions or simplifications.	Yes	Section 2.4, p.5; full study-level charting table in Supplementary File 2.
Critical appraisal	12	If done, explain why and describe how appraisal was conducted and used.	NA	Section 2.5, p.5: no formal risk-of-bias appraisal; rationale provided.
Synthesis of results	13	Describe methods of handling and summarising charted data.	Yes	Section 2.5, p.5: descriptive synthesis into three domains; outcome types interpreted separately.
RESULTS				
Selection of sources	14	Report numbers screened, assessed and included, with reasons for exclusions, ideally in a flow diagram.	Yes	Section 3.1, p.6; Figure 1: 270 identified, 44 duplicates, 226 screened, 69 full texts assessed, 25 reports/23 study families included.

Section/topic	Item	PRISMA-ScR checklist item	Reported	Location in manuscript
Characteristics of sources	15	Present charted characteristics for each source and provide citations.	Yes	Table 1, pp.16–18; Supplementary File 2.
Critical appraisal within sources	16	If done, present appraisal data.	NA	Not conducted; see item 12.
Results of individual sources	17	Present relevant charted data for each included source.	Yes	Sections 3.2–3.4, pp.6–8; Table 1, pp.16–18.
Synthesis of results	18	Summarise charting results in relation to the review questions and objectives.	Yes	Section 3.5, pp.8–9; Table 2, p.19.
DISCUSSION				
Summary of evidence	19	Summarise main results, link them to the review questions and consider relevance to key groups.	Yes	Sections 4.1–4.2, pp.9–10.
Limitations	20	Discuss limitations of the scoping review process.	Yes	Section 4.3, pp.10–11.
Conclusions	21	Provide a general interpretation, implications and/or next steps.	Yes	Section 4.4 and Section 5, p.11.
FUNDING				
Funding	22	Describe funding of included sources and of the review, including the funders' role.	Partial	Funding statement, p.12: review received no specific grant. Funding of included sources was not systematically extracted.

NA = not applicable. 'Partial' is used where the manuscript reports part, but not all, of the requested information.
